# Assessing the impact of storage time on the stability of stool microbiota richness, diversity, and composition

**DOI:** 10.1186/s13099-021-00470-0

**Published:** 2021-12-20

**Authors:** Elizabeth A. Holzhausen, Maria Nikodemova, Courtney L. Deblois, Jodi H. Barnet, Paul E. Peppard, Garret Suen, Kristen M. Malecki

**Affiliations:** 1 Population Health Sciences, School of Medicine and Public Health, University of Wisconsin–Madison, Madison, USA; 2 Department of Bacteriology, University of Wisconsin–Madison, Madison, USA; 3 Microbiology Doctoral Training Program, University of Wisconsin–Madison, Madison, USA

## Abstract

**Background:**

New technologies like next-generation sequencing have led to a proliferation of studies investigating the role of the gut microbiome in human health, particularly population-based studies that rely upon participant self-collection of samples. However, the impact of methodological differences in sample shipping, storage, and processing are not well-characterized for these types of studies, especially when transit times may exceed 24 h. The aim of this study was to experimentally assess microbiota stability in stool samples stored at 4 °C for durations of 6, 24, 48, 72, and 96 h with no additives to better understand effects of variable shipping times in population-based studies. These data were compared to a baseline sample that was immediately stored at − 80 °C after stool production.

**Results:**

Compared to the baseline sample, we found that the alpha-diversity metrics Shannon’s and Inverse Simpson’s had excellent intra-class correlations (ICC) for all storage durations. Chao1 richness had good to excellent ICC. We found that the relative abundances of bacteria in the phyla *Verrucomicrobia, Actinobacteria,* and *Proteobacteria* had excellent ICC with baseline for all storage durations, while *Firmicutes* and *Bacteroidetes* ranged from moderate to good. We interpreted the ICCs as follows: poor: ICC < 0.50, moderate: 0.50 < ICC < 0.75, good: 0.75 < ICC < 0.90, and excellent: ICC > 0.90. Using the Bray–Curtis dissimilarity index, we found that the greatest change in community composition occurred between 0 and 24 h of storage, while community composition remained relatively stable for subsequent storage durations. Samples showed strong clustering by individual, indicating that inter-individual variability was greater than the variability associated with storage time.

**Conclusions:**

The results of this analysis suggest that several measures of alpha diversity, relative abundance, and overall community composition are robust to storage at 4 °C for up to 96 h. We found that the overall community richness was influenced by storage duration in addition to the relative abundances of sequences within the *Firmicutes* and *Bacteroidetes* phyla. Finally, we demonstrate that inter-individual variability in microbiota composition was greater than the variability due to changing storage durations.

## Background

The human gut microbiome is a complex community of bacteria, viruses, and eukaryotes which aid in several vital functions including energy harvesting and storage, metabolism, stimulating maturation of immune cells, and development of healthy immune function [1]. Next-generation sequencing has led to the discovery of new bacterial species and the proliferation of studies investigating the role of the gut microbiome in human health, including population-based studies that rely on participant self-collection of stool samples. These population-based studies can take place across a wide geographic region, necessitating the shipment of stool to a central location for processing. Further, many of these studies rely on participants to self-collect samples in their homes.

An example of one such collection protocol was used by the Wisconsin Microbiome Study [2]. Briefly, participants were instructed to self-collect a stool sample in their homes within 24 h of a scheduled clinic appointment. Between sample production and the clinic appointment, participants were instructed to store the sample in their refrigerator (residential refrigerators are typically 4 °C). After the sample was collected at the clinic appointment, it was shipped on ice to a central location for further processing and long-term storage at − 80 °C. Thus, these samples were kept in cold storage for varying durations, potentially exceeding 24 h. This may be an important source of variability in population-based microbiome studies. To improve comparability and repeatability between large, population-based studies, better characterization of the effects of varying shipping times is needed.

Previous studies have investigated the effect of storage conditions on the stool microbiome with varying results [3–8]. Some have found that short-term storage at various temperatures did not have a substantial impact on the microbiome [3, 4], while others reported significant changes including loss of microbial diversity at room temperature and at 4 °C [5–8]. While these previous studies help characterize changes to microbiome samples that occur during storage, none replicate the conditions that samples undergo in a large, population-based or field study collecting microbiome samples over a large geographic area. Carroll et al. investigated the changes that occurred during storage at room temperature for up to 24 h, compared with a baseline sample and did not find significant changes in the relative abundances of taxa [3]. However, in studies where shipping of samples is necessary, sample storage times may exceed 24 h. In another study which investigated storage durations up to 14 days at room temperature, Lauber et al. concluded that there were no significant changes to the samples. However, this study did not include a baseline sample and compared 14 day-old samples to 3 day-old samples, and was unable to capture changes that may have occurred between sample production and 3 days [4]. Vogtmann et al. assessed changes to stool samples during storage, but relied on stabilizing solutions [5], which are not always appropriate in combination with stool self-collection methods, because the harsh chemicals may be harmful to participants if handled incorrectly. Thus, studies which depend on self-collection of fecal samples present an additional challenge to microbiome research, as sample collection, shipping, and storage protocols can induce additional variation. Further research is needed to characterize how these protocols may impact study outcomes.

The present analysis builds on previous research by assessing microbiota stability at 4 °C for storage durations of 6, 24, 48, 72, and 96 h with no additives. These conditions mimic storage and transit time of self-collected fecal samples to a laboratory, which is typical for large population-based or field studies. We aimed to quantify the impact of these shipping and storage conditions in order to provide recommendations for sample collection, shipping, and storage protocols.

## Methods

### Study participants and sample collection

The study was approved by the Health Sciences Institutional Review Board of the University of Wisconsin-Madison (#2016-0251). The study population consisted of 4 male and 8 female volunteers ranging in age from 22 to 55 years with with a mean age of of 35.4 years (SE 3.1). Volunteers were employees and students at the University of Wisconsin-Madison, Madison, WI. Participants completed a brief survey regarding age, gender, diet, and antibiotic use in the last 3 months. One participant reported eating a vegetarian diet, one reported a vegan diet, and one participant reported using antibiotics within three months prior to sample collection.

Volunteers were provided with a commode specimen collector (Fisherbrand Commode Specimen Collection System, Fisher Scientific, Hampton, NH) and were asked to document the time of stool collection and return the sample to the lab immediately after production. If the lab was unable to process stool sample within 30 min of production, the sample was not included in the study. Stool was mixed manually with a sterile plastic spatula and divided into 18 aliquots of 0.1 g under sterile conditions. Three aliquots from each sample were immediately frozen at − 80 °C to serve as a baseline (time 0). The remaining 15 aliquots (3 for each timepoint) per sample were stored at 4 °C for 6, 24, 48, 72, and 96 h before being transferred to a -80˚C freezer. Altogether, a total of 216 stool aliquots were analyzed.

### DNA extraction, PCR, and sequencing

The DNA extraction methods used in this analysis have been previously described in detail [2, 9, 10]. Briefly, bacterial cells were lysed mechanically using 0.1 mm diameter zirconia/silica beads protocol followed by enzymatic lysis with a cocktail composed of lysozyme, mutanolysin, lysostaphin, and SDS. DNA was extracted using phenol:chloroform:isoamylalcohol followed by isopropanol precipitation in the presence of sodium acetate. DNA was cleaned up using NucleoSpin Gel & PCR Clean-up Midi kit (Takara Bio USA, Inc., Mountain View, CA) and quantified on a Synergy 2 Multi-Mode Plate Reader (BioTek Instruments, Winooski, VT) using an Invitrogen Quant-iT PicoGreen dsDNA Assay Kit (Invitrogen, Carlsbad, CA). A total of 9 negative controls were inserted periodically after blocks of 24 samples. All negative controls yielded an undetectable amount of DNA. We did not find any effect of storing stool samples at 4 °C up to 96 h on DNA yield.

The gDNA was subjected to PCR amplification with primers targeting the V4 region of the16S rRNA gene, as previously described [11]. PCR products were purified on a 1% low melt agarose gel (National Diagnostics, Atlanta, GA) containing SYBR Safe DNA Gel Stain (Invitrogen, Carlsbad, CA). Bands of 380 bp were excised and purified using a Zymoclean DNA recovery kit (Zymo Research, Irvine, CA). Final DNA quantification was performed as described above and the resulting DNA from all samples were equimolarly pooled to construct a final sequencing library. Samples were sequenced on an Illumina MiSeq using a 2 × 250 bp paired-end v2 sequencing kit (Illumina, San Diego, CA), with a final library concentration of 10 pmol/l and 10% PhiX Control.

### 16S rRNA sequencing data processing

Raw sequencing data were processed using mothur [12] (version 1.43.0) software following the Standard Operating Procedure for MiSeq data [11]. Contigs (overlapping sequences) were aligned using SILVA [13] (v132version) and low-quality reads and chimeras detected by UCHIME [14] were removed. Sequences were assigned to operational taxonomic units (OTUs) with a threshold of 97% similarity using GreenGenes [15] (version gg_13_8_99) database. OTUs with less than 0.01% overall abundance within the dataset were considered rare OTUs and were filtered from the dataset. After rare OTUs were filtered, each sample was normalized to 15,000 reads.

### Statistical analysis

We analyzed stool samples provided by 12 individuals that were stored at 4 °C between 0 and 96 h before long-term storage at − 80 °C. At each time point (0, 6, 24, 48, 72, 96 h at 4 °C), we analyzed three replicates of the same specimen for each individual, resulting in a total of 216 samples. All alpha-diversity metrics and relative abundance measures were calculated using the phyloseq package in R [16]. To assess the stability of alpha-diversity measures over time, we compared the mean (by participant and storage duration) of observed OTUs, Chao1 [17], Shannon’s [18], and Inverse Simpson’s [19] and calculated the intra-class correlation coefficient (ICC) of each measure between each storage duration and baseline, accounting for repeated measures per individual. ICC metrics were calculated using the ICC package in R [20]. We interpreted ICCs as follows: poor: ICC < 0.50, moderate: 0.50 < ICC < 0.75, good: 0.75 < ICC < 0.90, and excellent: ICC > 0.90 [21].

We additionally assessed changes in microbial composition by examining how relative phyla-level sequence abundances changed over time for each of the top five most abundant phyla: *Firmicutes, Bacteroidetes, Verrucomicrobia, Actinobacteria,* and *Proteobacteria*. In this analysis, we compared the mean relative abundance of each phylum at baseline and each subsequent storage duration by calculating the ICC and accounting for repeated measures per individual. We used a square root transformation on each of the relative abundance measures to improve modeling assumptions.

Finally, to assess how beta-diversity changes with storage duration, we calculated the mean Bray-Curtis [22] dissimilarity indices between each individual and storage duration using the vegan package in R [23].

## Results

Among the triplicate samples from 12 individuals collected at 6 time points, sequencing of the V4 region of the 16S rRNA gene resulted in 11,135,458 total raw reads. After filtering of chimeras, low quality reads, and sequences of incorrect length, there were 8,695,917 remaining reads. Filtered reads were assigned to 3,744 unique OTUs at a 97% sequence similarity. The number of reads per sample ranged from 15,736 to 98,617 with a mean of 40,258 (SD 12,258), with an average of 120 (SD 17) unique OTUs.

**Fig. 1:**
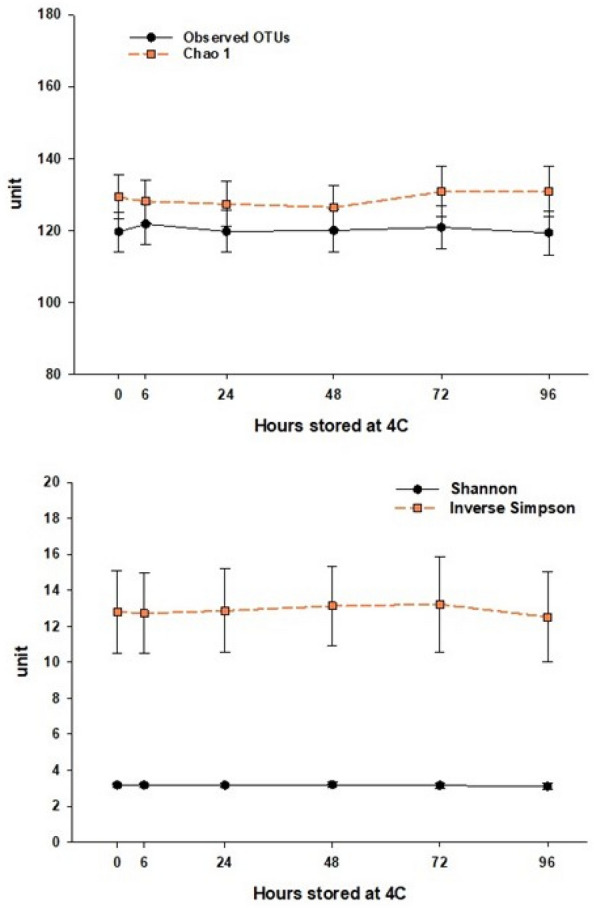
Mean alpha-diversity measures with 95% confidence interval of stool microbiota stored at 4 °C for between 0 and 96 h

To estimate the effect of storage time on microbiome richness and diversity, we compared several alpha-diversity metrics and the relative abundances of the five major phyla against a baseline sample, which was frozen at − 80 ˚C within 30 min of production (time 0). Figure 1 shows the mean and 95% confidence interval by storage duration of alpha-diversity measures observed OTUs, Chao1’s richness, Shannon’s, and Inverse Simpson’s. The mean observed number of OTUs fluctuated slightly with increased storage time, Chao1’s richness increased slightly, and Shannon’s and Inverse Simpson’s diversity remained relatively stable over time.

Table 1 shows the ICC of several alpha-diversity measures between baseline and samples subjected to different storage times. We found excellent intra-class correlation (ICC > 0.9) for Observed OTUs, Shannon’s diversity, and Inverse Simpson’s diversity at all time points. Chao1’s richness had good (0.75 < ICC < 0.90) to excellent intra-class correlation.

**Table 1:** Intraclass correlation coefficient (ICC) analysis of the mean alpha-diversity metrics in the microbiota of stool samples subjected to different storage times at 4 °C compared to baseline

Time	Observed ICC (95% CI)	Chao1 ICC (95% CI)	Shannon ICC (95% CI)	Inverse Simpson ICC (95% CI)
6 h	0.98 (0.94, 0.99)	0.91 (0.73, 0.97)	0.98 (0.93, 0.99)	1.00 (0.99, 1.00)
24 h	0.99 (0.95, 1.00)	0.89 (0.69, 0.97)	0.96 (0.88, 0.99)	0.97 (0.92, 0.99)
48 h	0.97 (0.90, 0.99)	0.87 (0.63, 0.96)	0.96 (0.88, 0.99)	0.96 (0.88, 0.99)
72 h	0.98 (0.92, 0.99)	0.88 (0.64, 0.96)	0.97 (0.89, 0.99)	0.96 (0.86, 0.99)
96 h	0.97 (0.89, 0.99)	0.86 (0.61, 0.96)	0.93 (0.79, 0.98)	0.94 (0.83, 0.98)

**Fig. 2:**
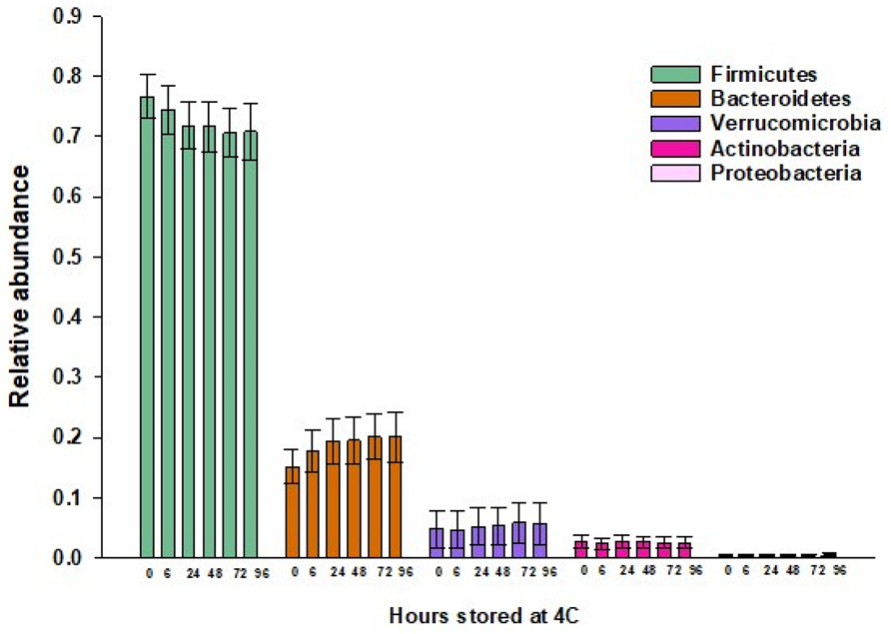
The effect of storage time at 4 °C on the relative abundances of the *Firmicutes, Bacteroidetes, Verrucomicrobia, Actinobacteria,* and *Proteobacteria* in the microbiota of stool

Next, we analyzed the relative abundances of microbial taxa at the phyla-level to assess how they were affected by storage time. Figure 2 shows the relative sequence abundance of the five major phyla by duration of storage. We found that the *Firmicutes* decreased in relative sequence abundance, while the *Bacteroidetes* and *Verrucomicrobia* increased as a function of prolonged storage time. In contrast, the relative sequence abundances of the *Actinobacteria,* and *Proteobacteria* were not substantially affected.

To further quantify how the relative abundance of the top five most abundant phyla changed over time, we calculated the ICC between baseline and our samples at different storage times, as shown in Table 2. The ICCs between baseline and subsequent storage times for the relative abundance of the *Firmicutes* and *Bacteroidetes* ranged from moderate to good. The ICCs between baseline and all subsequent storage durations for relative abundances of the *Verrucomicrobia, Actinobacteria,* and *Proteobacteria* was excellent.

**Table 2:** Intraclass correlation coefficient (ICC) analysis of the mean relative abundance of the top five most abundant phyla in the microbiota of stool samples subjected to different storage times at 4 °C compared to baseline

Time	Firmicutes ICC (95% CI)	Bacteroidetes ICC (95% CI)	Actinobacteria ICC (95% CI)	Proteobacteria ICC (95% CI)	Verrucomicrobia ICC (95% CI)
6 h	0.90 (0.70, 0.97)	0.93 (0.78, 0.98)	0.98 (0.92, 0.99)	0.98 (0.94, 0.99)	1.00 (0.99, 1.00)
24 h	0.72 (0.29, 0.91)	0.81 (0.48, 0.94)	0.98 (0.94, 0.99)	0.96 (0.89, 0.99)	0.99 (0.97, 1.00)
48 h	0.66 (0.19, 0.89)	0.77 (0.40, 0.93)	0.97 (0.90, 0.99)	0.96 (0.87, 0.99)	0.99 (0.96, 1.00)
72 h	0.66 (0.19, 0.89)	0.75 (0.35, 0.92)	0.97 (0.89, 0.99)	0.94 (0.82, 0.98)	0.98 (0.93, 0.99)
96 h	0.67 (0.20, 0.89)	0.78 (0.41, 0.93)	0.95 (0.84, 0.98)	0.95 (0.84, 0.98)	0.98 (0.93, 0.99)

**Fig. 3:**
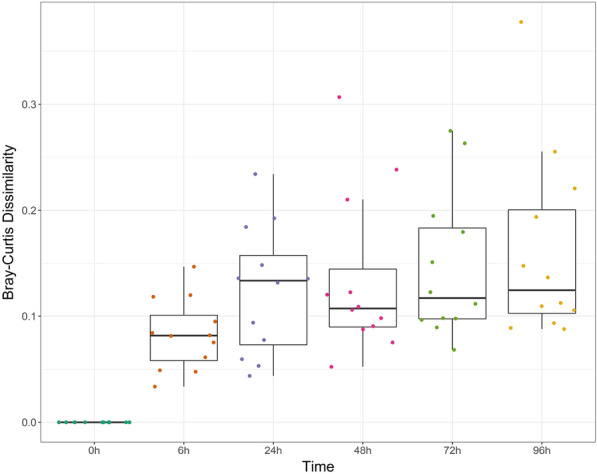
Mean Bray–Curtis dissimilarity index of the microbiota of stool samples subjected to different storage times at 4˚C, with error bars indicating the 95% confidence interval

We then calculated the Bray-Curtis dissimilarity index between baseline and subsequent storage durations to quanitfy the overall microbial community change as a function of storage time, as shown in Fig. 3. After 6 h, the median Bray-Curtis dissimilarity was 0.082 (IQR: 0.043). By 96 h, the median Bray-Curtis dissimilarity had increased to 0.12 (IQR: 0.098). We found that the largest change in microbial composition occurred within the first 24 h after stool collection, whereas between 24 and 96 h, the Bray-Curtis dissimilarity ranged between a minimum of 0.11 (IQR: 0.055) at 48 h and a maximum 0.13 (IQR: 0.084) at 24 h.

**Fig. 4:**
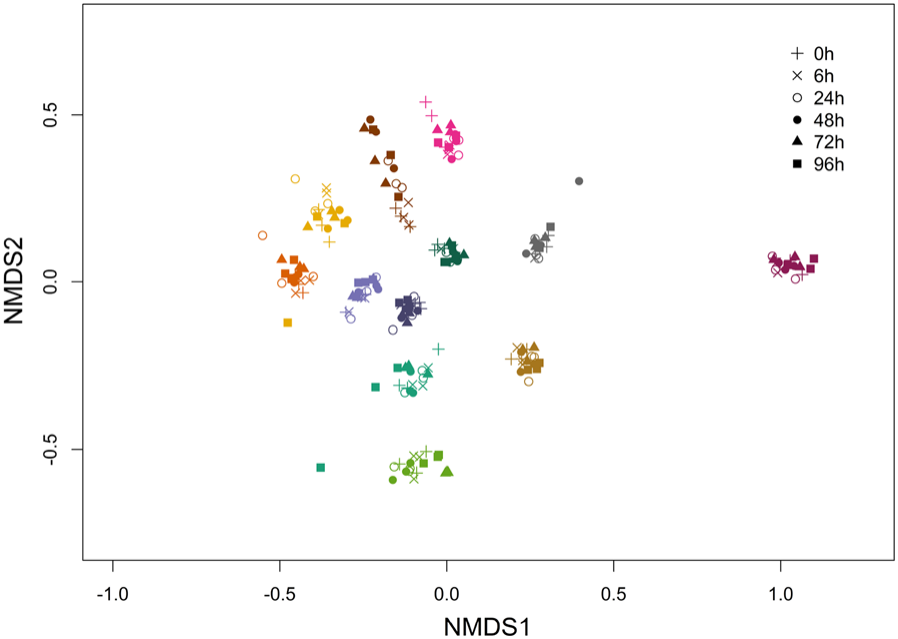
NMDS plot of Bray–Curtis distance matrices for all replicates of the microbiota of stool samples subjected to different storage times at 4 °C, with storage durations indicated by shape and colored by individual

Finally, we created non-metric multi-dimensional scaling plots using Bray–Curtis distance matrices, as shown in Fig. 4. Samples were found to strongly cluster by individual, suggesting that inter-individual variability was greater than the variability associated with storage time.

## Discussion

As gut microbiome research proliferates, it is increasingly important to understand the impact of sample processing protocols and conditions to ensure consistency, reproducibility, and reliability of study outcomes. We show that the greatest impact of storage time in changes to gut microbial composition occur within 24 h after sample collection, after which storage time up to 96 h does not change analytic results using standard diversity metrics. The Microbiome Quality Control (MBQC) and other studies have identified methodological differences in sample conditions, sample storage, and DNA extraction and sequencing technologies as key sources of variability among studies that may outweigh biological effects [24–26].

Sample shipping or transportation to the processing laboratory is often a major logistic consideration in field studies covering large geographical areas when immediate sample freezing is not possible or practical. In this study, we investigated the stability of the stool microbiota at 4 °C for up to 96 h which is a typical time frame between sample production and sample processing in the laboratory. We found that alpha-diversity metrics including observed OTUs, Chao1, Shannon’s and Inverse Simpson’s, were stable over time. When analyzing the concordance of microbial composition between the baseline sample (frozen within 30 min of production) and sample stored over different times, we found good to excellent correlation for Chao1 richness. For diversity metrics based on evenness and diversity, we found excellent correlation between baseline and all storage durations. Similarly, we found that the ICCs between baseline and different storage durations was excellent for the phyla *Verrucomicrobia, Actinobacteria,* and *Proteobacteria*. However, the ICCs for the *Firmicutes* and *Bacteroidetes* ranged between moderate to good as a function of storage duration.

The present findings extend those of Carroll et al., who compared stool samples that were stored at room temperature for up to 24 h against a baseline sample that was immediately frozen at − 80 ˚C and found that the microbiota was relatively stable even at room temperature [3]. While the present analysis employs the use of a baseline sample that was immediately (i.e. not more than 30 min after sample production) stored at – 80 °C, there is some evidence that changes to microbial composition can occur after 15 min of exposure to room temperature [8]. Limitations of this analysis were the small sample size and the inability to account for changes that may have occurred in the baseline samples prior to their storage at – 80 °C. The study relied on a convenience sample which may not be representative of the general population. However, because of the study design, each individual was their own control thus decreasing the probability that the results of this analysis were driven by confounding. The study sample, while small, still suggests that storage time from the field up to 96 h can maintain sample integrity, an important finding for planning future epidemiologic research.

## Conclusions

The aim of this analysis was to investigate the impact of storage duration at 4 °C on stool microbiota composition, to better understand the implications of varying storage and shipping times in large, population-based microbiome studies, especially the implications for between-study comparability and repeatability. Our analysis shows that measures of richness such as observed OTUs and Chao1 were impacted by storage time, as were the relative abundances of sequences in the phyla *Firmicutes* and *Bacteroidetes.* However, alpha-diversity metrics less sensitive to low abundance OTUs such as Shannon’s or Inverse Simpson’s diversity measures were largely unaffected by variable storage conditions. We also found that inter-individual variability in microbiota composition was greater than the variability due to storage durations. Therefore, the contribution of variability in microbiota composition due to shipping and storage times (less than 96 h) is smaller than variability due to biological differences between individuals.

## Data Availability

The datasets analyzed during the current study are available from the corresponding author upon reasonable request.
